# Challenges of teaching the deaf-blind learner in an education setting in Johannesburg: Experiences of educators and assistant educators

**DOI:** 10.4102/sajcd.v67i1.649

**Published:** 2020-06-11

**Authors:** Tejal Manga, Khetsiwe P. Masuku

**Affiliations:** 1 Speech Pathology, Faculty of Humanities, University of the Witwatersrand, Johannesburg, South Africa; 2 Speech Pathology and Audiology Department, Tembisa Hospital, Johannesburg, South Africa

**Keywords:** deaf-blindness, learners, educators, inclusive education, challenges, South Africa

## Abstract

**Background:**

Evidence suggests that educators of deaf-blind students in the South African context have specific challenges in the educational setting because of their lack of adequate knowledge on deaf-blindness and a lack of sufficient training on communication, teaching and learning strategies.

**Objectives:**

The aim of this study was to describe the challenges experienced by educators and assistant educators of children with deaf-blindness.

**Method:**

Ten educators and assistant educators were selected purposively to participate in the study (Male = 3; Female = 7; age range 31–49 years). Participants were recruited from a school for the deaf-blind in Johannesburg. Participants completed semi-structured interviews on the challenges that they experienced when educating learners who are deaf-blind.

**Results:**

Findings from the data after inductive thematic analysis suggested the following: (1) under-preparedness of educators and assistant educators, (2) communication challenges, (3) challenges related to the diversity of deaf-blind learners and (4) lack of support structures for educators and assistant educators.

**Conclusion:**

There is a need for ongoing educator training on communication strategies, cultural diversity and inclusive strategies. A collaborative model of delivering training and inclusive education that will encompass educators and therapists as a means of supporting both the educator and the learner who is deaf and blind is needed. Such a collaboration may result in positive outcomes for both the educator and the deaf-blind learner.

## Introduction

Deaf-blindness is a rare condition that affects approximately 1 million individuals worldwide. Many children who are deaf-blind are yet to be identified because of the complication of diagnosis owing to the comorbidities (DeafBlind SA, 2009). Even though the majority of the deaf and blind population in South Africa are adults, there still exists a percentage of children who present with deaf-blindness (DeafBlind SA, 2009). Deaf-blindness refers to a dual sensory impairment, which means that it is a combined condition of a loss of hearing and vision co-occurring in the same individual (Dammeyer, 2014; DeafBlind SA, 2009; Miles, 2008; Wittich, Southall, Sikora, Watanabe, & Gagné, 2013). It ranges from mild loss of hearing and vision to a complete deafness and blindness depending on its differing combinations (Ask Larsen & Damen, 2014) and it can either be congenital or acquired (Dammeyer, 2014; Molly, 2003a).

Persons who are deaf-blind may present with a diversity of needs across all areas of development, such as communication, parent–child relationships, cognition, motor and perceptual development (DeafBlind SA, 2009), which causes severe educational needs that cannot be accommodated in special education programmes solely for children with deafness or children with blindness (Wolford, 2016). Learning occurs through vision and hearing (Nelson & Bruce, 2016); therefore, learners who are deaf and blind need services that are provided by a team of skilled professionals and paraprofessionals who can create appropriate communication and learning opportunities for them (Riggio & McLetchie, 2008). Providing learners who are deaf-blind with appropriate communication and learning opportunities is imperative, because these learners depend on others to make language accessible to them and to provide them with the desire to communicate (Miles, 2008). There is a need for multiple communication strategies which should be implemented in both at the home and school environments to ease the communication challenges for both parties.

Section 29 (1) of the Constitution of the Republic of South Africa (1996); the Education White paper #6, on special needs education (Department of Education [DoE], 2001); Article 24 of the Convention of the Rights of Persons with Disabilities (CRPD) (United Nations [UN], 2006) and Sustainable Developmental Goals 4 and 10 (United Nations [UN], 2015) commit to provide equal educational opportunities to all learners, including those learners with special educational needs. These legislative documents make specific reference to those learners who experience barriers to learning and development or who have dropped out of the education system because of the inability of the education system to accommodate the diversity of their learning needs (DoE, 2001).

In South Africa, the provision of equal educational opportunities for all learners, and especially those with special educational needs, was operationalised through the drive for inclusive education. Inclusive education, a field that has been given importance by law, requires that educators are knowledgeable about policies and practice in inclusive education and that they are competent to teach in classes with diverse learners (Walton, 2018).

The White paper #6, in particular, acknowledges that educators are the primary resource for achieving the goal of an inclusive education and therefore commits to improve their skills and knowledge and to further assist them in facilitating the development of new skills (DoE, 2001). The White paper #6 further highlights the significant difference that schools can make if they were to provide a quality and relevant education for children with disabilities (DoE, 2001).

Challenges of implementing the White paper #6 in South Africa have been widely documented in the literature, such as in the works of Donohue and Bornman (2014), Lebona (2015), Kamper (2008), Engelbrecht and Green (eds. 2007) and Hay, Smit and Paulsen (2001). The gap that exists in the skills and training of educators of children with special education needs, specifically in inclusive education and in special schooling environments, has been highlighted as one of the significant challenges of inclusive education in South Africa (Dalton, Mckenzie, & Kahonde, 2012). The results of these teacher training and skills gaps are evident in the quality of care and engagements that deaf-blind individuals experience (Van Dyk, 2003). Because of the lack of knowledge and skills on communication, educators of deaf-blind students often miss or misinterpret the subtle, slow-paced and often difficult to understand interactions of these children, resulting in frustration for both the educator and the child with deaf-blindness (Van Dyk, 2003). Gaps in the implementation of the White paper #6, and ultimately inclusive education, therefore, have negative consequences for both the deaf-blind learner and the educator.

Within their formal basic teaching qualifications, mainstream educators do not receive, preparation on how to react within mainstream classrooms when teaching children with diverse learning needs (Ladbrook, 2009). Educators of children with special needs, including educators of learners who are deaf-blind, are therefore not exempted from these training gaps. Educators experience specific challenges (such as under-preparedness) to deal with learners presenting with multiple disabilities (such as deaf-blindness) because of their lack of adequate knowledge on different disabilities and the lack of training on communication, teaching and learning strategies (Charles, 2014; Janssen, Riksen-Walraven, &Van Dijk, 2003).

These findings were further echoed by Maguvhe (2014) in a South African study where the author investigated the perceptions of teachers of deaf-blind learners on curriculum design, implementation and parental involvement. Maguvhe (2014) reported that there was a need to establish effective training for educators of deaf-blind learners and to also establish unit standards with achievable outcomes for teaching learners with deaf-blindness, which was in line with the requirements of the South African curriculum.

The aforementioned challenges are further exacerbated in boarding school facilities where a wide diversity of the learners with deaf-blindness are cared for often by a limited number of staff for longer periods of time. South Africa, being a diverse country both culturally and linguistically, further contributes to the different ideas, not only about the needs of children with disabilities but also to best practices and beliefs regarding how they should be educated (Donohue & Bornman, 2014:3).

Traditionally, there has been a deprivation of adequate and appropriate support services for learners, teachers and schools (Barrat, 2016). There is, therefore, a need for a shift towards the inclusion of educational support services in special schools (Donald, Lazarus, & Lolwana, 2010), specifically to include services of speech therapists and audiologists in schools for learners who are deaf-blind. Speech therapists and audiologists may work with students who are deaf-blind, alongside each other to address communication issues because of sensory losses and additional disabilities (Rodriguez-Gil, 2009). This can be done by introducing strategies and interventions, such as alternative ways of communication with the deaf-blind learner. These will assist educators to cope with a diversity of learning and teaching needs to ensure that learning difficulties are addressed. In that way, the educator will be trained and supported.

International studies on the subject of deaf-blindness have focused on the communication challenges experienced by children with deaf-blindness and access to technology to increase communication access (Emerson & Bishop, 2012; Hersh, 2013), whilst others have highlighted the need for identification and intervention strategies for children who are deaf-blind (Anthony, 2016; Wiley, Parnell, & Belhorn, 2016). In South Africa, and Africa at large, deaf-blindness as a subject has generally been under-reported, with a plethora of research studies focussing on the implementation of inclusive education in schools for learners with special education needs. Deaf-blindness is one of those fields so often neglected, possibly because, as Maguvhe (2014, p. 1487) terms it, ‘it is a minority within a minority’, therefore making research in this area important and necessary. The lack of basic on-the-ground- services and support in the field of deaf-blindness is of more concern as it perpetuates the continued social exclusion of this population, such as institutionalisation, stigmatisation, isolation and lack of appropriate roles in society (Maguvhe, 2014), often with debilitating results. Ultimately, the rights of this population continue to be contravened despite the fact that the CRPD has explicitly declared the rights to access for all (UN, 2006). In particular, there is a paucity of research focusing on the training of educators of children who are deaf-blind.

The purpose of this article was to explore the challenges experienced by educators and assistant educators of children who are deaf-blind in a school specialising in the education of deaf-blind learners located in Johannesburg, South Africa. The specific research question was as follows: what are the experiences of educators of learners who are deaf-blind? This article argues that if educators of learners who are deaf and blind are provided with the necessary training on the skills and knowledge of facilitating communication with their learners, this would, in turn, contribute positively towards teaching and learning outcomes. Possessing the necessary skills of communicating with children who are deaf and blind would potentially lessen the frustration of both parties. Whilst educator training is a step in a positive direction, educator training alone would not be sufficient. Educators of and learners with deaf-blindness need to be provided with adequate educational support from communication specialists, such as speech-language pathologists and audiologists.

In addition to educational and communication support from speech-language pathologists and audiologists, educators need to be provided with the services of psychologists who will be able to provide them with emotional support and counselling.

## Methodology

### Research design

This study aimed to describe the challenges experienced by educators of learners who are deaf-blind in an education setting in Johannesburg. To achieve this aim, the study utilised an exploratory qualitative research design. Exploratory research design is a design of enquiry in which the researcher conducts a study in an area where there is a paucity of research already done (McMillan & Schumacher, 2001). This was an appropriate approach to use to study educators’ challenges of caring for the deaf-blind learners as the area of deaf-blindness is under-researched, possibly because of the low prevalence of the condition (DeafBlind SA, 2009). The study implored answers to the following questions: (1) describe the barriers of teaching learners who are deaf-blind; (2) ‘what are the facilitators of teaching learners who are deaf-blind?’ and (3) ‘what kind of support structures are available to educators of learners who are deaf-blind?’.

### Participants and setting

A purposive sample of 10 educators and assistant educators were included in the study. The ages of the participants ranged from 24 to 49 years, with the average age being 38.8 years.

Seven of the participants were women, whilst only three were men. Three participants had formal teaching qualifications in the form of a university degree, three had matric qualifications, whilst the remaining four had not completed their high school qualifications. Five participants had a working experience of 10 years and more, with the average work experience being 7.8 years. The study setting was a public boarding school situated in Johannesburg, South Africa, which specialises in the education of learners with deaf-blindness. The school has a total of 13 educators and educator’s assistants.

### Procedures and data collection

Prior to the commencement of data collection, ethical approval was obtained. All participants were provided with written consent forms for the interviews and for audio recordings before commencing with the interviews. There was no risk of harm to the participants. Anonymity and confidentiality were guaranteed and maintained. Participants were also informed of their right to withdraw at any point in the study if they felt uncomfortable, with no negative consequences.

Data were collected through semi-structured interviews. The interview questions focused on the experiences of teaching and caring for learners who are deaf-blind. All interviews were conducted in English and audio recorded using a digital recorder. Data were transcribed verbatim by the primary investigator. Transcripts were subsequently checked against the original data by the second author for accuracy.

### Data analysis

Data were analysed by the two authors using inductive thematic analysis, applying the procedures proposed by Braun and Clarke (2013), namely familiarisation, coding, searching for themes, reviewing themes, defining and naming themes and writing up. Ultimately, themes were constructed from patterns on the data sources. For consensus, we relied on a coding procedure to identify themes from words, phrases, sentences and paragraphs that represent or symbolise issues relating to experiences of the participants.

### Trustworthiness strategies

Trustworthiness strategies, such as credibility, transferability, confirmability and dependability, proposed by Shenton (2004) and Holloway and Wheeler (2013) were applied to the study. The primary investigator established a trusting relationship with the site and participants prior to the collection of the data. As investigators, we conducted frequent debriefing meetings during the entire process of the study. The project was subjected to peer reviews for scrutiny to ensure quality control. During interviews, member checking, probes and iterative questioning were applied. Furthermore, we kept a reflective journal to track the development of the study (eds. Chilisa & Preece, 2005; Shenton, 2004).

### Ethical consideration

Ethical approval to conduct the study was obtained from the Human Research Ethics Committee (HREC) of the University of the Witwatersrand (project number STA_2017_09), the Department of Speech Pathology and Audiology Sub-Committee, University of the Witwatersrand and Department of Education.

## Results and discussion

Four themes emerged from the analysis of the data collected: (1) under-preparedness of educators and assistant educators, (2) communication challenges, (3) challenges related to the diversity of deaf-blind learners and (4) lack of support structures for educators and assistant educators. These themes are discussed in detail below.

### Theme1: Under-preparedness of educators and assistant educators

Because of the lack of education and training on teaching leaners with deaf-blindness, a majority of participants reported being under-prepared in their role of teaching the learners. In response to whether educators and assistant educators had any history of and training or education on teaching deaf-blind learners, participants shared the following:

‘The time when I first got here – it was a very, very difficult time for me because I didn’t have any idea or any experience in teaching learners who are deaf-blind. I didn’t have any idea about sign language and things like that …’ (24 years old, female, teaching assistant)

‘The first one [*challenge*] is that we don’t have training. No workshops either are provided to us. No training was provided.’ (49 years old, male, teacher)

‘If they can give us more time … in terms of more time for us to learn about these disabilities … they usually give us two or three days and sometimes that is not enough.’ (30 years old, male, teaching assistant)

‘I have never had any training except that I went for observations to Worcester in 2006 … So I never went to any College or University … but maybe it’s because I also didn’t know if there is any course to do with the deaf-blindness that is available to capacitate us.’ (49 years old, female, teacher)

Current findings support the narrative that educators, assistant educators and staff working in schools catering for children with deaf-blindness are not adequately equipped with the necessary skills and knowledge to teach and care for children with deaf-blindness. These impact the teaching, guidance and communication support that they provide to the children with deaf-blindness (DeSimone & Parmar, 2006; Maccini & Gagnon, 2006). The lack of training on deaf-blindness results in teachers believing that they are not adequately prepared to instruct learners with deaf-blindness (DeSimone & Parmar, 2006), which is often frustrating for them. Teachers find themselves in situations where they have to take on the task of teaching, caring and supporting these children, regardless of the lack of preparedness; consequently, they resort to educating themselves on deaf-blindness (Gendreau, 2011).

### Theme 2: Communication challenges

Participants in this study experienced challenges communicating with learners who are deaf and blind.

These communication challenges are attributed to the lack of skills necessary for educators to facilitate communication between themselves and the learners. Participants particularly struggled with skills and knowledge for facilitating tactile as a means of communication. Furthermore, they reported difficulty in interpreting information communicated to them by the learners. Participants recounted using their previous experiences of what worked, with other deaf-blind learners, to improve current communication skills.

This finding is expressed in the following quotes:

‘He can’t say I need the water or I am hungry or I need to go bathroom. I have to just think that he needs something and that I should give her water, then maybe she doesn’t want that water, then take her to the toilet, then she doesn’t want to go toilet … it’s a challenge because I don’t know what is going on … I wish to know what is going on but I can’t know and I just have to assume.’ (31 years old, female, assistant educator)

‘I was never taught how to communicate with them.’ (49 years old, male, educator)

‘It is a barrier because even though they can’t see, they can’t talk, they can’t hear, you still have to teach them sign language... But they won’t understand like … it will take time for them to understand.’ (45 years old, male, assistant teacher)

‘Just something to motivate us, not to say we are stressing them. As I said maybe they aren’t talking but me, I am a human being and sometimes I don’t know what is going on, so sometimes they will cry really … like the whole day and I don’t know what to do what’s going on with them.’ (31 years old, female, assistant educator)

As a result of the communication challenges, learners who are deaf-blind expressed their frustration through the display of challenging behaviours such as tantrums, as be seen in the following quote:

‘My interpretation of what they want can be wrong. So when I do something maybe the child will throw tantrums because I am not responding to what he or she wants from me.’ (24 years old, female, educator assistant)

Frustration as a result of communication barriers was also experienced by some educators and assistant educators, as evident in the following quotes:

‘At first I thought I was in another world when I first came here, and even now I sometimes still feel this was because ultimately you are speculating and searching and you have to do the searching until you find what the problem you must guess what he wants is, it does become frustrating.’ (49 years old, female, educator)

‘I want to know is how can... how can I communicate with these types of people, how I can talk, how can I see when he/she is cross … How can I.’ (43 years old, female, assistant educator )

‘“Ultimately you are speculating and searching and you have to do the searching until you find what is the problem” and that, “you must guess what he wants” it can get hard.’ (24 years old, female, teaching assistant)

The findings of the study confirmed that the ability to foster a means of communication with a deaf-blind child is both the most important and the most challenging requirement (Miles, 2008). This is particularly true in school settings where educators lack training on communication strategies (Charles, 2014). The obvious difficulty for the person who is deaf-blind in trying to communicate is that very few people understand their communication attempts (eds. Aitken, Buultjens, Clark, Eyre, & Pease, 2000). Consequently, the lack of an effective communication system interferes with and hinders activities of daily living for the child with deaf-blindness (Omugur, 2016). Communication challenges in children who are deaf-blind, coupled with difficulties with self-regulation and self-monitoring, may trigger frustration and result in challenging behaviours in this group (Greg, 2017; Nelson & Bruce, 2016). Communication challenges can further cause frustration also for the communication partners (Greg, 2017), who in this case happen to be the educators and assistant educators of these children. It is therefore imperative that an effective method of communication is fostered to prevent misinterpretations between the two parties (Gendreau, 2011). This can be established by training educators and assistant educators on deaf-blindness, communication strategies and facilitating communication.

### Theme 3: Diversity of deaf-blind learners

South Africa is home to a variety of cultures. It is a culturally diverse country with many different teachings, values and practices that are unique to each culture. This diversity was also noted in the learners at the study site. Learners presented with diversity in terms of their age, family, social, religious and cultural backgrounds. This diversity presented a challenge for educators and assistant educators as it had implications for the type and level of sign language to which learners are exposed, the level of support that learners receive from their families and the acceptable way of engaging with learners undergoing adolescence. This is captured in the following statements:

‘In terms of culture, because the deaf-blind children cannot express themselves so because of this lack of expression the diversity of the culture sometimes restricts us as the educators … There are times when the child will get irritated in the class room and the culture does not allow the teacher to assist that child – so as a teacher what must I do? So that’s why I am saying the diversity regarding the culture is a barrier because the children come from different cultural backgrounds.’ (49 years old, female, educator)

‘Ay it’s really difficult because they are from a different environment and as we are here and we have to try to make them feel at home here. They are from different homes and different upbringing and here we must take care of them as if they are from one family, you see.’ (49 years old, male, educator)

‘“It’s so painful”… even their parents … some of them can just say “go to the school and I don’t want to know what is happening” because they are feeling shame and when the child is at home they will just lock him or her in the room and so the mother can go up and down and the people they can’t see the kid, you see.’ (43 years old, female, assistant educator)

The findings of this study support the notion that the rich diversity of South Africa contributes to the different theories about the needs of children with disabilities, as well as best practices and beliefs regarding how they should be cared for and educated (Donohue & Bornman, 2014, p. 3). Whilst diversity is acknowledged and appreciated, it however presents challenges for educators of deaf-blind children, especially in a boarding school facility. Omugar (2016) asserts that much of the work of educators of deaf-blind teachers is mired by exterior influences. Factors such as different family, social, cultural environments and varied moods of the students make it difficult for educators to cope with their levels of sign language and communication needs (Omugar , 2016). It is a well-known fact that in special educational contexts, children often vary in terms of age, which makes it difficult for teachers to cope with specifically knowing how to deal with those deaf-blind learners going through adolescence (Omugar, 2016).

### Theme 4: Lack of support structures for educators and assistant educators

The majority of educators and assistant educators in this study had no basic formal teaching qualifications; furthermore, they had not been capacitated and supported to deal with children presenting with deaf-blindness. Educators and assistant educators were not provided with support that they needed in terms of workshops, training, counselling and debriefing, as evident in the following quote:

‘Not in South Africa … at all … Nothing in South Africa. Nothing for deaf-blind. No one in South Africa assisted us with workshops or training.’ (49 years old, female, educator)

In response to being questioned about receiving counselling and/or debriefing, one participant replied as follows:

‘When the schools are closed, we get days off, we can get somebody who can come in to counsel us because sometimes when things are happening, you can say it’s an easy thing but in your mind we are feeling something bad and anger and … you see, those things.’ (43 years old, female, assistant educator)

The findings of this study arecorroborated by other studies that have emphasised the need for educators and assistant educators to be supported if children with special educational needs, such as deaf-blindness, are to succeed in the education setting (Bornman & Rose, 2010; Dalton et al., 2012; Frankel, Gold, & Ajodhia-Andrews, 2010; Nel et al., 2016). Sustenance and care that educators capable of offering to learners depend on the knowledge, skills and resources provided to them (Nel et al., 2016).

Therefore, if teachers lack the necessary knowledge, skills and resources, they will not be able to fulfil the necessary roles in adequately supporting children with deaf-blindness. If the goal of inclusive education for children with disabilities is ever to be realised, educators need to be provided with new skills, training and support, which will enable them to appreciate and address the variety of diverse learning needs of these children (Dalton et al., 2012; Frankel et al., 2010).

## Implications for inclusive education support systems

The role and influence of educators and assistant educators as teachers, communication partners and carers of children with deaf-blindness cannot be overemphasised. Communication is central to deaf-blindness and therefore it is imperative that a communication method that is understood by the educator and peers of the deaf-blind child is established early in the child’s life. A functional communication method is imperative in the deaf-blind learner’s establishment of relationships, facilitation of social participation, facilitation of independence, access to social activities and, most importantly, in their progress across their education trajectories.

Establishing a functional communication method, and ultimately the access to education, for children with deaf-blindness will not be possible if the lack of knowledge, skills, preparedness and support on the part of educators and assistant educators continues as the norm in deaf-blind schools. The lack of proper training amongst teachers of children with deaf-blindness is one of the major hindrances to the education of these children; therefore, it needs to be addressed as a matter of urgency.

The Department of Education (DoE) therefore needs to train educators and assistant educators and capacitate them through ongoing workshops and courses on disability, specifically deaf-blindness, communication strategies and facilitating communication with these children. The DoE must also realise the challenges associated with being a teacher and a carer for a deaf-blind child and establish support structures in the form of peer support, counselling and debriefing. Assistant educators play a vital role in the education of pupils with complex learning disabilities, routinely supporting students on a 1:1 basis without the direct supervision of teachers. Therefore, there is a need to also include them in the training that is provided for educators.

## Conclusion

The findings of this study suggest that communication is at the centre of the learner–educator partnership for the deaf-blind learner. Therefore, if the right support is provided to educators, assistant educators and carers of deaf-blind children, learners would thrive in their educational contexts. Children who are deaf-blind also have the right to access teachers who have specific knowledge and training on deaf-blindness and also to information and resources specific to their needs. Training needs to cut across the following areas: knowledge training on disability and specifically deaf-blindness, skills development on managing children with deaf-blindness and communication strategies for the deaf-blind, and it should be considerate to the individual cultural and linguistic characteristics of the learner. Providing constant support to educators and assistant educators of deaf-blind students would also ease the external challenges negatively affecting their role. Therefore, there is a need for a collaborative model of delivering inclusive education that will encompass educators, therapists and families of children with deaf-blindness as a means of supporting both the educator and the learner who is deaf and blind. Such collaboration will potentially result in positive outcomes for both the educator and the deaf-blind learner.

## Data Availability

Data sharing is not applicable to this article as no new data were created or analysed in this study.
